# Advances in human papillomavirus detection for cervical cancer screening and diagnosis: challenges of conventional methods and opportunities for emergent tools

**DOI:** 10.1039/d4ay01921k

**Published:** 2024-12-23

**Authors:** O. Fashedemi, Okoroike C. Ozoemena, Siwaphiwe Peteni, Aderemi B. Haruna, Leshweni J. Shai, Aicheng Chen, Frankie Rawson, Maggie E. Cruickshank, David Grant, Oluwafunmilola Ola, Kenneth I. Ozoemena

**Affiliations:** a Advanced Materials Group, Faculty of Engineering, The University of Nottingham Nottingham NG7 2RD UK oluwafunmilola.ola@nottingham.ac.uk; b Department of Chemistry, University of Guelph Ontario Canada; c Molecular Science Institute, School of Chemistry, University of the Witwatersrand Johannesburg 2050 South Africa kenneth.ozoemena@wits.ac.za; d Department of Biomedical Sciences, Tshwane University of Technology Pretoria 0001 South Africa; e Aberdeen Centre for Women's Health Research, University of Aberdeen Aberdeen AB25 2ZD UK

## Abstract

Human papillomavirus (HPV) infection is the main cause of cervical cancer and other cancers such as anogenital and oropharyngeal cancers. The prevention screening and treatment of cervical cancer has remained one of the top priorities of the World Health Organization (WHO). In 2020, the WHO came up with the 90–70–90 strategy aimed at eliminating cervical cancers as a public health problem by the year 2030. One of the key priorities of this strategy is the recommendation for countries to ensure that 70% of their women are screened using a high-performance test by the age of 35, and again by the age of 45. Over the years, several traditional methods (notably, Pap smear and nucleic acid-based techniques) have been used for the detection of cervical cancer. While these methods have significantly reduced the incidence of cervical cancer and death, they still come short of excellence for the total eradication of HPV infection. The challenges include low sensitivity, low specificity, poor reproducibility, the need for high-level specialists, and the high cost of access to the facilities, to mention a few. Interestingly, however, several efforts are being made today to mitigate these challenges. In this review, we discussed the pros and cons of the traditional screening and testing of HPV infections, the efforts being made to improve their performances, and the emergent tools (especially, the electrochemical methods) that promise to revolutionize the screening and testing of HPV infections. The main aim of the review is to provide some novel clues to researchers that would allow for the development of high-performance, affordable, and triage-suitable electrochemical-based diagnostic tools for HPV and cervical cancer.

## Abstract 2

This review evaluates various point-of-care (POC) methodologies for HPV detection and highlights the current advancements in POC diagnostic devices.

## Graphical abstract

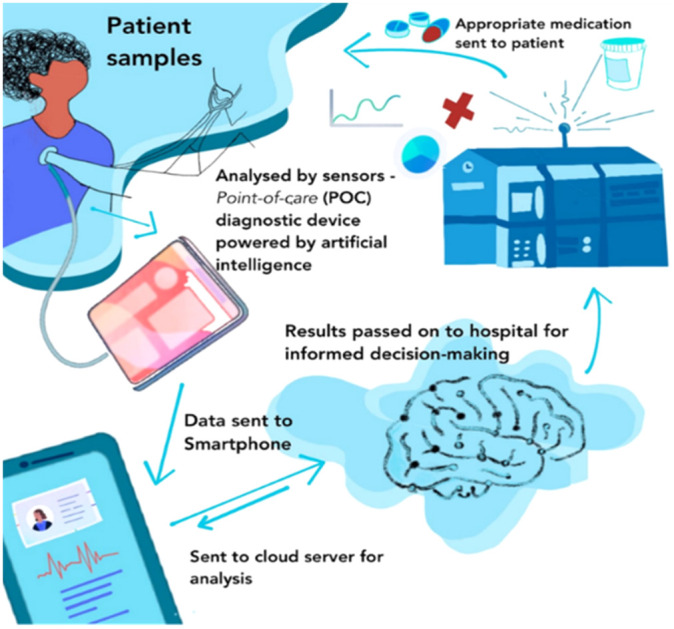

## Introduction

Persistent human papillomavirus (HPV) infection is the leading cause of cervical cancer. Cervical cancer remains the fourth most common cancer in women (after breast cancer, colorectal cancer, and lung cancer). According to the 2018 report,^1^ there were about 570 000 cases and 311 000 deaths arising from cervical cancer, mostly occurring in resource-limited countries in Sub-Saharan Africa, Asia, and Latin America. In sub-Saharan Africa, cervical cancer is the leading cause of cancer-related deaths in women. For example, Eswatini was reported as the country with the highest incidence, with about 6.5% of women developing cervical cancer before the age of 75 years.

As part of the strategies to curb the incidence of cervical cancer and death, in August 2020 the WHO adopted the so-called 90–70–90 target for 2030: (i) 90% of girls to be fully vaccinated with the HPV vaccine by the age of 15, (ii) 70% of women screened using a high-performance test by the age of 35, and again by the age of 45, and (iii) 90% of women with pre-cancer treated and 90% of women with invasive cancer managed.

**Fig. 1:**
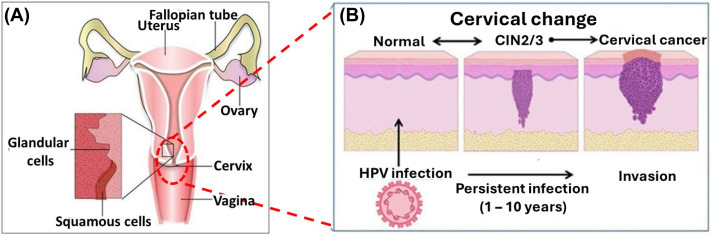
(A) Pictorial depiction of the cervix anatomy^5^ (B) schematic illustration of the cervical changes caused by HPV infection.^6^

All HPV genomic structure consists of a circular, double stranded DNA. Nearly all HPV have three distinct regions *i.e.* the long control region (LCR) or “L-region” which does not code for any proteins, the early region also known as the “E-region”, this encodes 6 viral proteins (E1, E2, E4, E5, E6, E7), E6 and E7 encodes two viral proteins which are oncogenic and lastly the late region (L-region), the L-region encodes structural proteins, L1 which is the major capsid protein and L2 the minor capsid protein. These proteins participate in the genomic replication, transcription, cell cycle, cell signalling and apoptosis control, immune modulation and structural modification of HPV infected cells.^2,3^ Of the 14 high-risk HPV types, two of them (HPV-16 and HPV-18) are considered the most important as they have been observed in 62% of cervical cancers. Some literature states that HPV-16 and HPV-18 are responsible for up to 70% of cervical cancer cases, with HPV-16 present in about 60% of cases and HPV-18 in about 10%. HR-HPV encode specific viral genes, such as E6 and E7, which have been associated with cervical cancer cell lines. These viral genes produce oncoproteins that can be integrated into the hosts genome, such as E6 and E7 proteins. The E7 protein binds to retinoblastoma family proteins, impeding the inhibition of transcription factors and leading to uncontrolled cell cycle progression. Consequently, E7 protein degrades pRB function leading to an overexpression and disruption of the epithelial cell cycle, therefore leading to uncontrollable growth of cancerous cells.^4^ HPV infection persistence can result in precancer changes, known as cervical intraepithelial neoplasia (CIN) which is curable if detected by screening when dysplastic cells are confined within the surface epithelium of the cervix. Cervical intraepithelial neoplasia (CIN) is in three stages: CIN1, CIN2, or CIN3. If left untreated, CIN2 or CIN3 (collectively referred to as CIN2+) can progress to cervical cancer as schematically shown in Fig. 1.^5–8^ These have the potential to penetrate the basement membrane to become invasive cervical cancer and spread in nearby organs *e.g.*, the uterus, bladder, rectum, and pelvic lymph nodes, thereby causing death. Apart from cervical cancer, hr-HPV are the causative agent of other cancers such as the anal, vulvar, penile, head and neck cancer.^9–12^ Thus, early detection with low-cost and sensitive diagnostic devices will greatly benefit low-income areas. Precise and time-efficient measures could improve early diagnosis and treatment.

In Europe, cervical cancer remains the second most common cause of death in women aged between 15 and 44 years with reported diagnoses and death totalling 61 072 and 25 829, respectively. The incidence and mortality rates were estimated at 15.9 and 6.7 per 100 000 women in Europe, respectively, in comparison with a mortality rate of 6.8 per 100 000 women globally.^13^ In the UK, the highest incidence rates were observed in young women between the ages of 25–29 years.^14^ Vaccination and screening have the potential to reduce the incidence rate of cervical cancer to 4 per 100 000 women globally.^13^ Currently, there are 6 types of HPV vaccines that are approved by the FDA, namely 2vHPV (bivalent vaccine), 4vHPV (quadrivalent vaccine), and 9vHPV (9-valent vaccine), of the bivalent type, there are 2 vaccines and of the quadrivalent type, there are 3 vaccines. As their names suggest, the bivalent HPV vaccine can only be used for HPV 16 and 18, the quadrivalent can only be utilised for HPV 6, 11, 16 and 18, while the 9-valent HPV vaccine can be used for 6, 11, 16, 18, 31, 33, 45, 52, and 58. Although the HPV vaccination programmes are meant to curb and control the progression of high risk HPV to cervical cancer cases, LMICs are still burdened with poor vaccination rollouts, this can be contributed to a number of factors such as high cost of vaccine acquisition, lack of storage infrastructure for vaccines, logistics *etc.* This, therefore, necessitate for development of cost effective, highly precise methods for early screening of HPV.^15,16^ Also, screening aims to reduce cervical cancer incidence and mortality by the early detection of precancerous lesions that can progress to invasive cancers if undetected and untreated. Regrettably, the uptake of cytology-based screening (*i.e.*, Pap smear test) has fallen in recent years in the United Kingdom, with population coverage of 77.8% achieved in 2014 compared to 80.6% in 2004.^17^

In many low-income and middle-income earning countries (LMICs), no organized cervical cancer screening programs exist. Resourced screening is rarely offered because there is limited infrastructure to support current screening approaches using cervical cytology. In these countries, the detection of cervical cancer and precancer is mainly based on direct visual inspection. One advantage of the visual screening approach is the direct possibility of treating suspect lesions in the same session, the ‘Screen and Treat’ approach.^18^

Strategies for cervical cancer prevention and control in LMICs require concerted efforts to improve screening and access to treatment, especially in high-risk HIV populations. LMICs bear the largest burden of human immunodeficiency virus (HIV) infection while persistent high-risk HPV infection is more common among HIV-infected women. Thus, the risk of cervical cancer is increased in women with HIV/AIDS. HIV clinics provide opportunities to ‘screen and treat’ cervical precancer and cancer in this population.^18,19^ This highlights the need for point-of-care HPV molecular diagnostics for a test and treat model in high-risk HIV populations. Implementation of the traditional Papanicolaou (Pap) smear in national screening programs is not sustainable in under-resourced LMIC settings with a limited skilled cytologist workforce^20^ and where, despite a high prevalence of cervical cancer, lack of follow-up and poor adherence to treatment are major impediments for program success. The current World Health Organization (WHO) recommendation for HPV testing as a primary cervical cancer screening tool has been adopted by several countries such as Kenya, where it forms part of the national cancer screening guidelines.^21^ However, it is difficult to detect small ectocervical and endocervical lesions under visual inspection (as in the case of Pap smear procedures). Depending on the screening intervals, lesions might be overlooked and develop into invasive cancer. On the other side, many changes observed in visual inspection of the cervix with acetic acid (VIA) are rather non-specific and can result lead to substantial overtreatment of women. Treatment risks include haemorrhage, infection, obstetric complications including premature birth, cervical stenosis with failure to progress in labour and uterine rupture if not recognized, and increased susceptibility toward transmission of other sexually transmitted diseases (STDs), especially HIV.^22^

Besides high morbidity and mortality, another serious problem of cancer is the widening of socioeconomic inequalities, with the most notable gaps for the most preventable cancers. For example, compared with the most affluent counties, mortality rates in the poorest counties were 2-fold higher for cervical cancer and 40% higher for male lung and liver cancers during 2012–2016.^23^

Current tumour diagnosis relies on various complex clinical settings, including X-ray imaging, computerized tomography (CT), magnetic resonance imaging (MRI), positron emission tomography, endoscopy, sonography, thermography, cytology, and biopsy. In addition, molecular tools based on both genomic and proteomic are increasingly used, such as polymerase chain reaction (PCR), enzyme-linked immunosorbent assay (ELISA), radioimmunoassay (RIA), immunohistochemistry (IHC), and flow cytometry.^24–26^ Of the existing technologies, most of them are invasive, expensive, time-consuming, and limited to laboratory centres in large hospitals.

Protein and nucleic biomarkers from body fluids such as tears, urine, sweat, saliva, and blood have been widely used in diagnosis and prognosis. Exosomes on the other hand are nano-sized bio vesicles released into surrounding body fluids upon the fusion of multivesicular bodies and the plasma membrane. They were shown to carry cell-specific cargos of proteins, lipids, and genetic materials, and can be selectively taken up by neighbouring or distant cells far from their release, reprogramming the recipient cells upon their bioactive compounds. Therefore, the regulated formation of exosomes, the specific makeup of their cargo, and their cell-targeting specificity are of significant biological interest. They are considered non-invasive diagnostic biomarkers, as well as therapeutic nanocarriers. As exosomes can be released by practically all eukaryotic cells, it is thought that their cargos may greatly differ from each other for the function of the originated cell types and their current state (*e.g.*, transformed, differentiated, stimulated, and stressed). Thus, exosomes and their biologically active cargo may offer both diagnostic and prognostic information in a range of diseases, such as chronic inflammation,^27^ cardiovascular and renal diseases^28,29^ neurodegenerative diseases,^30^ lipid metabolic disease,^31^ and tumours.^32^

This review investigates the different cancer biomarkers and techniques for detecting these biomarkers. The different point-of-care approaches for HPV detection and the current level of development of (POC) diagnostics devices are highlighted. However, a major concern with the existing HPV screen-and-treat approach is the overtreatment of high-risk HPV-positive women. The review also explores other innovative point-of-care molecular diagnostic tools that are sensitive and specific using biomarkers that can serve both diagnostic and prognostic purposes in HPV screening which can be integrated into primary health settings in LMICs as screen-and-treat models. These point-of-care platforms can further be used to diagnose multiple conditions and monitor therapy on a single device.^33^

## Conventional techniques for HPV detection

**Fig. 2:**
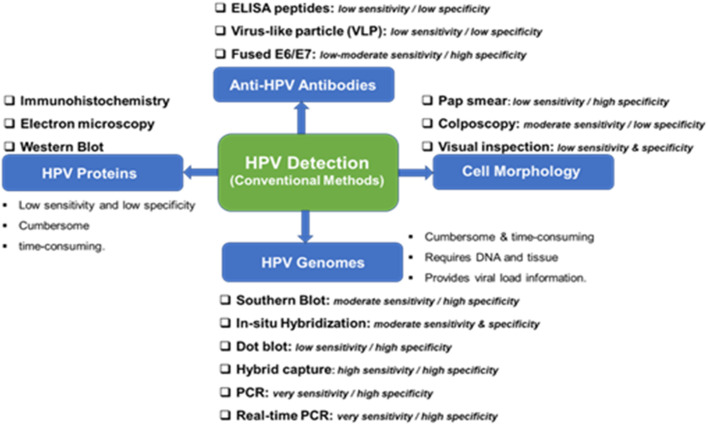
Conventional methods from the detection of HPV and their clinical sensitivity and specificity for CIN 2/3 lesions and cervical cancer.

The conventional methods for the detection of HPV are well documented in^34,35^ and may be categorized into four (Fig. 2): (i) detection of the morphology of the cell, (ii) detection of HPV genomes, (iii) detection of HPV proteins, and (iv) detection of anti-HPV antibodies. Morphological changes in the cervix (*e.g.*, precancerous, and cancerous lesions) arising from HPV infections are detected using the Papanicolaou stain (Pap smear), colposcopy, or by visual inspection analysis. Cytology or Pap smear represents the most successful HPV prevention worldwide as it has led to a significant reduction in cervical cancer and death. However, the main clinical drawbacks of the Pap smear are its low sensitivity, low specificity, and poor reproducibility.

It is not possible to culture HPV, so its accurate detection has mostly relied on molecular biology. The genome of HPV is well known to encode two early proteins (E1 and E2) that perform regulatory functions, two early proteins (E6 and E7) that carry out regulatory functions, and two late capsid proteins (L1 and L2). The role of protein E4 is not known, but protein E5 is hydrophobic and thought to improve cell immortalization. As shown in Fig. 2, the use of HPV genomes for the detection of HPV infection and cervical cancer involves the use of the Southern blot analysis which is the gold-standard method for HPV genomic analysis. Southern blot methodology is time-consuming and requires significant amounts of purified DNA. Other methods in this category are *in situ* hybridization, hybrid capture, and real-time polymerase chain reaction (RT-PCR). Conventional PCR-based diagnostic tools are characterized by their high reliability, sensitivity, and specificity for the detection of HPV genotypes. Generally, these HPV genomes methodologies are characterized by their technical difficulty, time-consuming, and the need for DNA and tissue preservation. However, it provides information on the viral load.

Immunological-based detection of HPV is frustrated by certain challenges: (i) L1 and L2 are only expressed at the productive HPV infection stages; (ii) the early proteins (notably, the E1, E2, E6, and E7) proteins are expressed at very low levels in HPV infected cells, and (iii) the inability to produce high-quality antibodies that are sensitive and specific to the target HPV proteins. The detection of HPV proteins involves the use of immunohistochemistry (detection of L1 and L2 in squamous intraepithelials), electron microscopy, and western blot. In general, the detection of HPV proteins is characterized by low sensitivity, low specificity and can be cumbersome and time-consuming. The fourth method involves the detection of anti-HPV antibodies including ELISA peptides, the detection of virus-like particles (VLP), both of which have low sensitivity and specificity, and fused E6/E7. The pros and cons of these conventional detection techniques are summarized in Fig. 2.

### Some recent efforts at improving the conventional HPV detection techniques

#### PCR method

Real-time polymerase chain reaction uses extracted DNA samples that are subject to a real-time polymerase chain reaction (PCR) using gene-specific primers, which target specific segments of the HPV genome. In some modified PCR-based methods, messenger RNA molecules (mRNAs) resulting from the transcription of genes E6 and E7 are reverse-transcribed into complementary DNA, which in turn, is amplified in a PCR reaction. Oncoproteins E6 and E7 are the major drivers of oncogenesis in infected individuals, and thus their overexpression, detected at either the mRNA or protein level, is predictive of the risk of developing cancer or may indicate oncogenesis, regardless of the absence or presence of lesions. E6 and E7 also drive tumor transformation. Melting curves are analysed using the analysis program provided with the thermocyclers. Weak positivity of HPV results, when analysed using PCR, are reported as negative results, therefore the need for an electrochemical method, which gives specific results. Alternative, to real-time PCR, which is prone to several short coming, droplet-digital PCR (ddPCR) is a promising technique with improved sensitivity, elevated detection rates and, does not require the use of standard calibration *etc.* The ddPCR technique relies on the use of limited PCR volumes and Poisson statistics. As a basis for this technique, samples are diluted and partitioned in multiple reaction chambers or droplet. The absolute quantity of PCR fragments is quantified from volumetric water-in-oil droplet partition using the same standard PCR primers and fluorescence probes, the absolute concentration is calculated using Poisson statistics. This method has been used to simultaneously detect and determine a numerous number of HPV genotypes. Lv *et al.* reported on the use of ddPCR for multiple determination and quantification of HPV genotype among the Chinese population, and the results showed high sensitivity, specificity and accuracy compared to the traditional PCR [N]. In another study reported by Lillsunde Larsson *et al.* ddPCR for a series of high-risk HPV was studied, and later, ddPCR efficacy was compared with traditional PCR for HPV 16 only and ddPCR showed superior efficacy of 31 : 1 of traditional PCR. These studies show the superiority of ddPCR compared to traditional PCR.^36–39^ However, the use of PCR methods for the detection of HPV is both labour-intensive and cumbersome with the need for a highly skilled technical expert for the interpretation of obtained results. In addition, PCR tests do not provide evidence of past infection therefore the natural course of the disease cannot be studied.^39–41^ The use of first void (FV) urine to test for HPV antibodies has shown that there is “a good correlation between HPV 6, 11, 16 and 18-antibodies in FV urine and paired sera, as well as between both assays”, which confirmed that HPV-Abs originating from CVS are detectable in FV urine of young women, although at low levels.^42^ This inadequacy limits the detection of the existing risk of developing cervical cancer, especially in settings characterized by low vaccination rates and limited prior HPV screening.

#### DNA microarray

This is a laboratory tool used to detect the expression of thousands of genes at the same time. DNA microarrays are microscope slides that are printed with thousands of tiny spots in defined positions; each spot contains a known DNA sequence or gene known as gene chips or DNA chips. Each DNA molecule attached to each slide acts as a probe to detect gene expression (transcriptome) or messenger RNA (mRNA) transcripts expressed by a group of genes. First, mRNA molecules are collected from both an experimental sample (an individual with a disease like cancer) and a reference sample (a healthy individual). The two mRNA samples are then converted into complementary DNA (cDNA); each sample is labelled with a fluorescent probe of a different colour *e.g.* (cDNA) green, or fluorescent dye while the experimental cDNA sample is a red fluorescent dye. The two dyes are mixed for binding to occur which is known as hybridization; it is then measured to determine the expression of each gene printed on the slide. For the gene that is expressed higher than the other, the colour will be high *e.g.*, if it is experimental, the colour will be green but if both show equal expression, then the spot will appear yellow. The final data gathered can be used to create a gene expression profile in response to a particular treatment of the condition.

The susceptibility of the dye used in microarrays to the ozone effect makes it unstable in the presence of ozone which lowers the signal strength and prevents the scanner from recognizing it. Although it takes only a few minutes to convert the raw signal data of microarray into high-quality data for further processing,^43^ further analyses can be done using different in-house software packages such as PePPER, FIVA, DISCLOSE, PROSECUTOR.^44–47^ This is followed by result verification which can be done using qPCR or β-galactosidase assays using *lacZ* promoter fusions.

Gogianu *et al.*^48^ reported the use of carbon dots for the first time to increase DNA microarray biochips' detection performance, the authors achieved a fluorescent hybridization intensity of 3.744 for HPV 16. Recently, Varesano *et al.*^49^ combined the use of HPV 16/18 genotyping and microRNAs detection, as a triage test for HPV-positive women to identify subjects at high risk for cancer progression.

#### Immunology-based method

Three serological assays commonly used in clinical trials for the detection of HPV antibodies are HPV pseudovirion-based neutralization assay (PBNA), competitive or total Luminex immunoassays (cLIA or LIA) and VLP-based enzyme-linked immunosorbent assays (ELISA).^50^ The gold-standard method is PBNA which has the following drawbacks: difficult to set up, laborious, and does not discriminate between different antibody isotypes and subclasses. As for cLIA/LIA and VLP-ELISA, they have high throughput and are rapid; but their reagents and equipment are difficult to source. However, the standard good laboratory practices which sometimes give a false positive result in the laboratory must be addressed especially when performing analysis of samples from the same individuals on the same plate as, the pre-and post-samples, this will eliminate both inter-plate variations and appropriate controls *i.e.*, pooled serum controls.^41^ Furthermore, the traditional antibody-based methods may not be sensitive enough to detect the infection in the early stages, especially because the expression of viral proteins may be low during this period, requiring a more sensitive method for the detection of low-abundance proteins. The antibody-based detection methods of E6 and E7 mRNAs hybrids with specific DNA probes have been developed and reported. The authors revealed that the method detects E6 and E7 mRNA without the costly amplification of nucleic acids, rather with the use of the S9.6 antibody and horseradish peroxidase (HRP)-linked secondary antibody.

## Emergent techniques for HPV detection

The limitations of the conventional HPV detection techniques have informed the need to explore other techniques that would allow for sensitive, specific, fast detection, and low cost. Some of the emergent diagnostic tools include the Fourier transform infrared spectrophotometry (FTIR), Raman spectroscopy and surface-enhanced Raman scattering (SERS), mass spectrometry, and electrochemical methods. As the readers will see later, of all these methods, electrochemical methods have received much attention.

### Fourier transform infrared spectrophotometry (FTIR)

Fourier transform infrared spectroscopy can potentially improve clinical decision-making and patient outcomes by detecting biochemical changes in cancer patients at the molecular level. It is more simple, more rapid, more accurate, inexpensive, non-destructive, and suitable for automation compared to existing screening, diagnosis, management, and monitoring methods.

There are three regions for the infrared spectrum: near-infrared (NIR) in the 0.76–2.5 μm (12 500–4000 cm^−1^) region, mid-infrared (MIR) in the 2.5–25 μm (4000–400 cm^−1^) region, and far-infrared in the 25–1000 μm (400–10 cm^−1^). The most used region for biological applications is MIR, which consists of the fingerprint region of 1800–900 cm^−1^ for proteins (amide I/II/III), lipids, carbohydrates, and nucleic acids. NIR spectroscopy may be used in similar applications to MIR spectroscopy. NIR spectra are occupied by overtone (resonant bands above the fundamental bands) and combinational bands with the typical absorption coefficients two orders of magnitude lower than that of MIR fundamental bands. Therefore, NIR light can penetrate much deeper into the sample surface than MIR light, which makes NIR spectroscopy better suited for deep tissue sampling and the examination of highly moist specimens.

FTIR records spectrochemical information composed of the absorption intensities for each wavenumber of the mid-infrared spectrum (4000–400 cm^−1^).

**Fig. 3:**
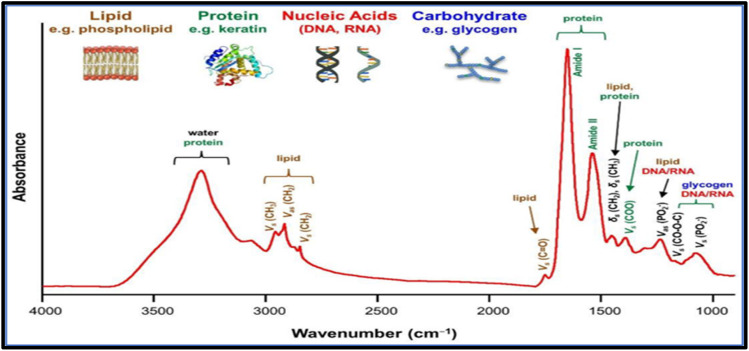
Common Fourier transforms infrared (FTIR) bands for biomolecules.^51^

The infrared bands carry vibrational information used to identify the molecular components and their respective structures; thus, the spectra generate a distinctive molecular fingerprint used to screen and scan samples in various segments. The fingerprint spectrum for biological samples also called the “bio fingerprint” region ranges from 1800 to 900 cm^−1^ as shown in Fig. 3 (ref. 51) and contains information on key biomolecules such as lipids, proteins, carbohydrates, and nucleic acids^52^ Changes in the IR signature for these biomolecules are associated with concentration changes (changes in band intensity) and changes in molecular configuration and neighbouring functional groups (band shifts towards higher or lower wavenumbers). Thus FT-IR generates chemically rich spectral signatures of tissue or biofluids that can be used for a wide range of clinical applications, especially in oncology.^53^

**Fig. 4:**
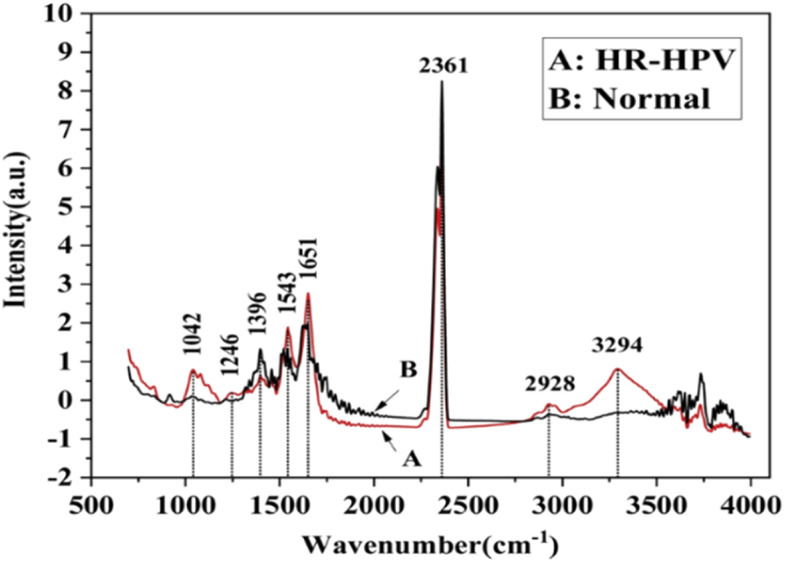
Normalized mean FT-IR spectra of HR-HPV positive patients and normal people (the red solid line (A) is the FT-IR spectra of cervical exfoliated cells of 50 HR-HPV positive patients, and the black solid line (B) is the FT-IR spectra of cervical exfoliated cells of 50 normal people).^60^

FTIR has been reported as a non-invasive method for the detection of HPV.^54–61^ For example, Mo *et al.*^60^ employed FTIR as a non-invasive method to detect HR-HPV in patients. A total of 100 spectra were recorded from 50 HR-HPV-positive patients and 50 normal subjects. They found clear differences in the recorded spectra of the two groups (Fig. 4). Unlike the normal subjects, the spectra of the HR-HPV patients showed peaks at the 1042 cm^−1^ (mucin), 1246 cm^−1^ (amide III), 1396 cm^−1^ (proteins), 1543 cm^−1^ (amide II), 1651 cm^−1^ (amide I), 2361 cm^−1^ (CO_2_), 2928 cm^−1^ (lipids), and 3294 cm^−1^ (amide A).

The sensitivity of FTIR spectroscopy to chemical changes during the transition from normal to pathological state or during treatment can lead to the identification of novel biomarkers associated with the disease,^62,63^*in situ* chemical composition analysis of cirrhosis by combining synchrotron Fourier transform infrared and synchrotron X-ray fluorescence spectroscopy on the same tissue section.

However, some authors^57,58,61^ have suggested FTIR spectroscopy be considered a complementary diagnostic method to PCR, cytology, immunofluorescence, molecular histopathology, and other methods.

### Raman spectroscopy and surface-enhanced Raman scattering (SERS)

**Fig. 5:**
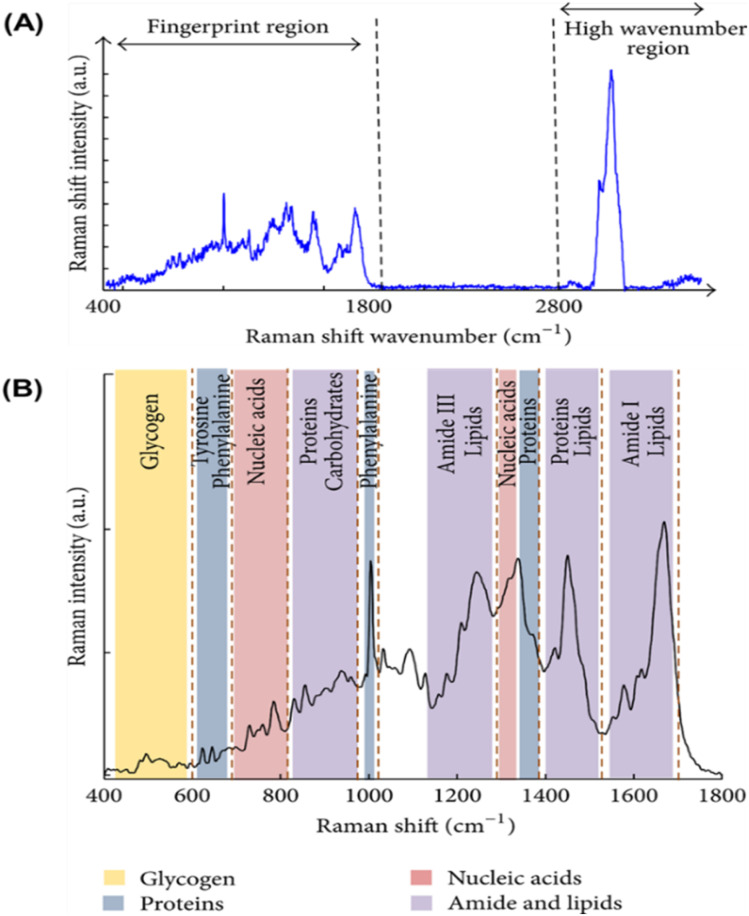
(A) Raman spectrum of cervical cancer CaSki cell line. The variation of Raman shift wavelength is expressed in wavenumbers (cm^−1^) and can be observed along the *X*-axis whilst the intensity is represented along the *Y*-axis. The fingerprint and the high wavenumber (HW) regions of the spectrum are indicated by the arrows. (B) Fingerprint region of the Raman spectrum of cervical cancer CaSki cell line. The major assignments related to glycogen, proteins, lipids, and nucleic acids are highlighted.^64^

Raman spectroscopy has emerged as an important technique for the detection of various types of pathologies, including cervical cancers.^64–73^ It has been used in understanding the progression of the disease at the molecular level. The Raman spectrum of any sample is acquired by simply irradiating the sample with a laser source (of either visible or near-IR monochromatic irradiation) and then measuring the scattered radiation with an appropriate spectrometer. Since every molecule exhibits highly specific and distinctive spectral features, a Raman spectrum can serve as a crucial identification marker for a particular sample. Fig. 5A depicts a typical full Raman spectrum of a cervical cancer cell line, CaSki.^64^ It shows characteristic features in the fingerprint (400–1800 cm^−1^) and high wavenumber (2800–3500 cm^−1^) regions. Fig. 5B is the expanded fingerprint region, highlighting the major assignments that are associated with glycogen, proteins, lipids, and nucleic acids. Surface-enhanced Raman scattering (SERS) is an ultrasensitive analytical vibrational spectroscopic technique. The enhanced SERS signal is attributed to the combined electromagnetic and chemical effects. The ability to generate high Raman signal by this technique is mostly dependent upon the SERS-active substrates used, most preferably the bimetallic nanomaterials such as gold–silver (Au–Ag) materials. Many researchers have adopted bimetallic Au–Ag nanomaterials for SERS because of the inherent advantages of generating stronger and sharper surface plasmon resonance and excellent SERS activities compared to the monometallic counterparts. Recently, Ning *et al.*^74^ reported the ultrasensitive detection of HPV-16 using specific oligonucleotides based on Au@AgAg bimetallic nanorods.

### Clustered regularly interspaced short palindromic repeats (CRISPR)

Clustered regularly interspaced short palindromic repeats (CRISPR)-based assay is an emerging technology that has found application in the sensitive detection of pathogens.^75–86^ CRISPR is a gene editing technique, that can be used to edit genes with mutation or be used to cure some diseases. CRISPR consists of two important components: the guide RNA (gRNA) and Cas9 protein. The gRNA acts as a guide by identifying DNA target while Cas9 protein cuts the identified DNA target. The gene editing consists of several essential steps. Firstly, the gRNA identifies the target DNA, after which the Cas9 protein cleaves the targeted DNA. This is followed by the repairing step at the target site through the non-homologous end joining (NHEJ) or homology-directed repair (HDR) pathways. Two approaches can be used for gene editing *i.e.* the single and multiplex approach. In the single approach, the single guide RNA directs the Cas protein to a single target DNA where it binds and cleaves the target DNA after which editing of the gene occurs, while the multiplex approach involves the sgRNA identifying multiple target DNA, and the Cas protein cleaving multiple DNA targets, after which simultaneous DNA editing occurs.^87–89^ A new strategy that involves the combination of CRISPR and SERS has also emerged and proving to be a hot topic due to its extraordinary sensitivity.^77,90–93^ For example, Choi *et al.*^94^ reported the use of the CRISPR/SERS system to enhance the detection of HPV-16 and HPV-18 to an extremely low detection limit (*i.e.*, ato-molar concentration level) at a very short time of 20 minutes. Su *et al.*^95^ CRISPR/Cas-SERS to detect HPV genes in serum with a very low detection limit in the pico-Molar concentration level.

CRISPR/Cas SER-based technology holds great promises in HPV diagnosis, and as attested by recent authors,^96–98^ its good sensitivity and specificity need to be further explored in complex biological environments using minimally invasive samples such as biofluids (blood, urine) or exfoliated cells on larger patient cohorts.

### Mass spectrometry

Mass spectroscopy (MS) determines the molecular mass of a charged particle by measuring its mass-to-charge (*m*/*z*) ratio. A mass spectrum is a plot of ion abundance *versus m*/*z*. A mass spectrometer consists of an ion source that converts molecules to ionized analytes, a mass analyser that resolves ions according to the *m*/*z* ratio, and a detector that registers the number of ions at respective *m*/*z* values. The mass analyser depends on three key parameters: sensitivity, resolution, and mass accuracy. The sensitivity, resolution, and accuracy of advanced mass spectrometers allow the detection of femtogram levels of individual proteins in complex mixtures. As recognized by the 2002 Nobel Prize in Chemistry, innovation of electrospray ionization (ESI) and matrix-assisted laser desorption/ionization (MALDI) techniques has made it possible to ionize big molecules such as proteins, peptides, and nucleotides for mass spectrometric analysis. ESI generates ions at atmospheric pressure by injecting a solution-based sample through a small capillary. MALDI produces ions by pulsed-laser irradiation of a sample that is co-crystallized with a solid matrix that can absorb the wavelength of light emitted by the laser. Protonation or deprotonation is the main source of charging for the ions generated in ESI/MALDI.^99^

Mass spectroscopy imaging, MSI is mostly used in cancer research and consequentially for oncogenic viruses. Different MSI-based techniques, alone or in combination with various other proteomic and imaging approaches have been successfully applied to study virus infections, virus-induced tumours, and antiviral compounds. It provides molecular information on a large variety of analytes including their spatial distribution. Therefore, this label-free imaging approach is a promising option for the analyses of tissues and cells in biomedical research.^100^ Schwamborn *et al.*^101^ reported the combination of traditional morphology analyses and cytological evaluation with MALDI MSI-derived molecular signatures to facilitate the automated diagnosis and stratification of high-risk HPV-derived cervical carcinomas into different cervical Papanicolaou (Pap) classes using cytospin preparations of Pap smears. The authors were able to screen and classify cervical cytology samples and simultaneously diagnose HPV infections routinely and in an automated manner using this feasible approach. Godoy-Vitorino *et al.*^102^ recently reported a study on the detection of HPV + H infections in urine, *via* analysis of some urine metabolites using just 200 μL of the urine sample. The metabolite separation was done by gas chromatography and detection by mass spectroscopy. The study provided preliminary evidence for the successful detection of urine metabolites related to cervical high-risk HPV infections. The GC-MS analysis showed that patients with high-risk HPV infections have a significantly higher abundance of 5-oxoprolinate, erythronic acid, and *N*-acetylaspartic acid. Besides characterizing cervical HPV, the authors were able to relate high-risk HPV infections with urinary metabolites and defined 5-oxoprolinate, erythronic acid, and *N*-acetylaspartic acid as possible prognostic biomarkers for high-risk HPV infections.

MS-based proteomics analyses of complex protein mixtures have also been reported.^103^ They usually require a starting amount in the range of 0.1–10 μg, depending on the experimental setup and the type of mass spectrometer used. In contrast to other standard techniques, MSI is neither restricted to one or more defined analytes nor limited by the availability of antibodies, fluorescent chromophores, or nucleic acid probes. Furthermore, MSI techniques are highly versatile and specific at the same time, as numerous masses can be simultaneously detected and discriminated.

### Electrochemical methods

**Fig. 6:**
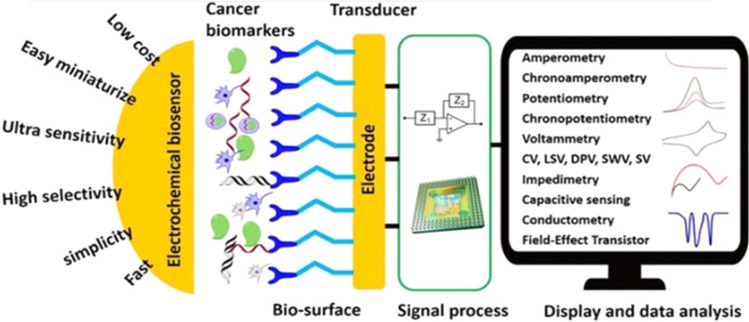
Schematic diagram of advantages, component parts, and various measurement methods of electrochemical biosensor.^104^

Electrochemical biosensors are very important alternative tools to other detection methods for pathogens, including cervical cancers. As shown in Fig. 6,^104^ electrochemical biosensors are characterized by several unique advantages, including fast response time, simple to use, low cost, easy to miniaturize, highly sensitive, and selective. These advantages are important for cervical cancer detection. For example, the ability of the electrochemical biosensors to give an ultra-low limit of detection is crucial because the concentration of the cancer biomarkers is very low in their early stages.^105^ A typical electrochemical biosensor comprises two main components. The transducer is made up of two components: the bioreceptors and the interface (transducer). Both serve as recognition probes for target analytes. Table 1 also shows commonly adopted methods for the detection of HPV.

**Table 1:** Summary of traditional methods used for HPV detection^112^

	Detection method	Pros	Cons
DNA level	PCR	Simple, quick sensitive highly specific	Easy to cross-contamination between samples high false positive rate perform HPV typing operations is tedious
	DNA ISH	High specificity can be used for localization detection	Detection is affected by the DNA content in the specimen results are sometimes difficult to explain
mRNA level	RT-PCR	High sensitivity high specificity accurately reflects the transcription activity of viral oncogenes	Requires technical expertise testing fresh frozen tissue
	RNA ISH	High sensitivity high specificity to some extent, it can be used as the “gold standard” for HPV-related tumors	The experimental operation requires high experience poor repeatability
Protein level	p16 immunohistochemistry	High sensitivity strong prognostic indicators	Low specificity mainly used for the detecting HPV in oropharyngeal cancer
	IHC	Virus antigen detection easy to perform and locate	False-negative findings and low sensitivity

Some recent developments in electrochemical biosensing include the discovery of bioreceptors with high specificity and affinity, the design of novel redox tags to conduct multiplex bioassay and ratiometric electrochemical assay, the development of signal amplifiers based on nanomaterials, and the integration of electrochemical biosensors with microfluidic chips.^106,107^ The receptor is the most important component for the design of an electrochemical biosensor, which involves antibodies, lectins, peptides, deoxyribonucleic acid (DNA), peptide nucleic acids (PNAs), aptamers, molecularly imprinted polymers (MIPs).^104,108^ It is the biosensing element to which the analyte has a highly specific binding affinity. Antibodies (immunoglobulins) are immune system-related proteins, which can selectively bind to antigens with a high binding constant of more than 10^8^ L mol^−1^. The significant advantage of antibodies is the specificity and affinity of these probes to target analytes.

Electrochemical biosensors can provide fast, accurate, sensitive early detection, measure and analyse the effectiveness of anticancer chemotherapy drugs in a non-invasive style and monitor cancer metastasis and angiogenesis. It will be a strong candidate for cancer theranostics because it possesses the advantages of high selectivity, low cost, ultra-sensitivity, simplicity, easier of be miniaturized, and mass fabrication which grant them a better fit for point-of-care (POC) devices at home or clinic.^109^ Such a method can be developed based on an antibody reacting with either the E6 or E7 oncoproteins, whose detection can reveal the level of risk for developing HPV-induced cervical cancer. The method was previously reported for the detection of cholera, based on an antibody-probe system detecting cholera toxin as the target antigen.^110^ It is therefore plausible to presume that such a method can be adapted for the diagnosis of HPV infection directly *via* the detection of HPV-derived oncoproteins, or indirectly by detection of host proteins affected by interaction with these oncoproteins, such as tumour suppressor p53 and cell cycle checkpoints. The latter strategy may only give evidence of the extent of the infection and the development of cancer.

**Fig. 7:**
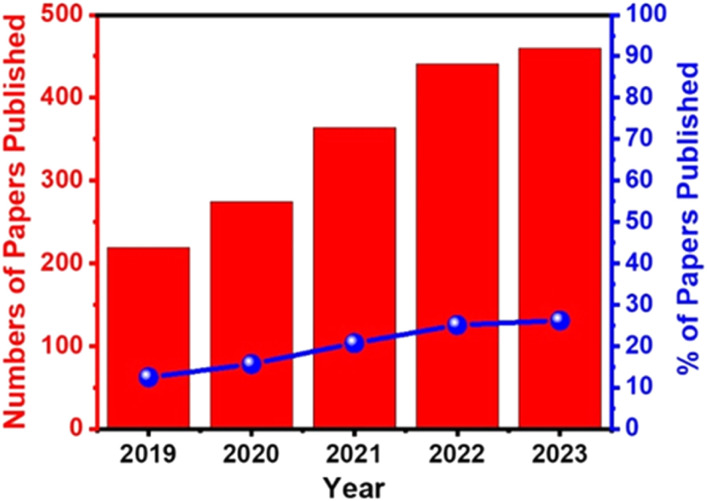
Percentages of papers published on electrochemical detection of cervical cancer/HPV from 2019 to 2023 (Google Scholar database, keywords “electrochemical analysis of cervical cancer, electrochemical detection of HPV”).

Electrochemical biosensors play a crucial role in the development of point-of-care (POC) diagnostics because they are simple to use, rapid, real-time, cost-effective, and easy to miniaturize and mass-produce. They also can be used as point-of-care (POC) devices at home or doctor's office. Therefore, they have received significant attention, and extensive efforts have been devoted to developing ultrasensitive electrochemical biosensors for the detection of cancer markers with high selectivity.^111^ For example, the publication rate in electrochemical detection of HPV grew steadily from 2019 to 2023 (Fig. 7).

**Fig. 8:**
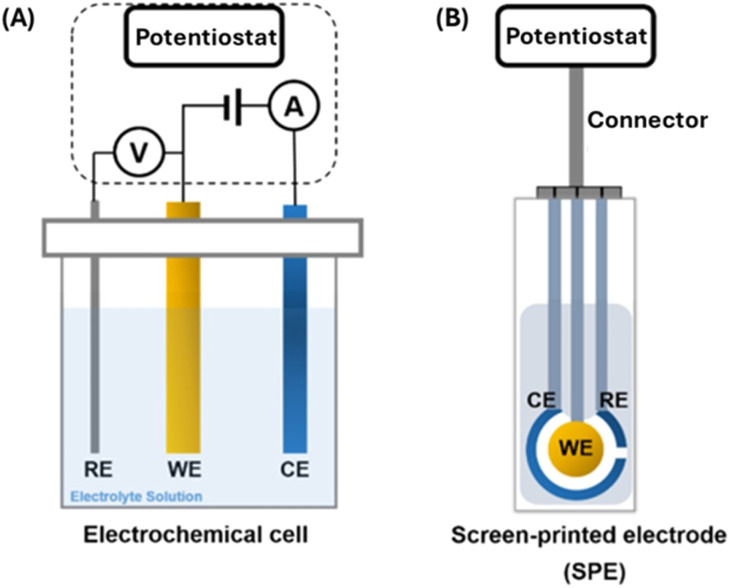
Electrochemical sensors – three electrodes system (A) electrochemical cell (B) screen printed electrode (SPE).^113^

The electrochemical biosensor^113^ measurement depends on the impulsive interaction between a chemical reaction and electrical energy that involves an oxidation–reduction reaction to generate an electrical current or *vice versa*. The chemical process that occurs between immobilized biomaterials and the analytes caused the production/consumption of ions or electrons, that affect the electrical current, the electrical potential, or any other electrical property of the solution. These reactions occur at a metal/semiconductor electrode and an electrolyte interface. Thus, detection is feasible if the reactions occur in close contact with the electrode surface. Hence, the electrodes significantly influence the performance of the electrochemical biosensor. One must consider several factors when choosing a proper electrode, including its material, dimension, and possibility to carry out surface modifications. Most electrochemical cells are composed of three electrodes (Fig. 8 (A)): (1) a reference electrode (RE), which is usually Ag/AgCl and positioned at a distance from where the reaction takes place. This is to provide a potential that is proportional to the known and stable solution. Furthermore, the RE allows normalization of the measurements. (2) A counter electrode (CE), also known as auxiliary electrode is the source of the current that is afterward applied to the working electrode. (3) A working electrode (WE) which is the sensing or reduction/oxidation electrode. The WE acts as the transducer in the biochemical reaction. The CE and WE should be chemically stable and conductive. Therefore, the main electrode materials used are gold, silver, platinum, silicon, carbon, and graphene depending on the analyte and the nature of the reaction. The screen-printed electrode (SPE) (Fig. 8 (B)) is an alternative to the electrochemical cells used in electrochemical sensors. The SPE is a version where the three electrodes are screen printed on an insulating substrate. The SPEs have several advantages of simplicity, scalable, low-cost and low analyte/reagent consumption.

Antibody-based electrochemical biosensors, (electrochemical immunosensors) are one of the most common biosensors for cancer protein biomarkers detection.^114,115^ Lectins are natural proteins of non-immune origin with specific binding affinity for the glycan moiety of glycolipids and glycoproteins. They are valuable recognition elements for the biosensing of glycoprotein tumour markers.^116^ Peptides are short chains of amino acid monomers linked by amide bonds. They represent a promising class of biorecognition elements that can be coupled to electrochemical transducers. Compared to antibodies, they are more stable in harsh environments and selectivity toward a target analyte. Furthermore, they can be synthesized easily and modified with specific functional groups, thus making them suitable for the development of novel architectures for biosensing platforms. Peptides have also been proposed as anti-biofouling agents.^117^

Deoxyribonucleic acids (DNAs) are stable, low-cost, and easily adaptable molecules. They have been employed to build a variety of biosensors *via* the interactions between DNAs and biomolecules or chemical compounds with high sensitivity and selectivity. DNA-based label-free electrochemical biosensors, without additional assay reagents and tedious procedures, have also attracted tremendous attention from researchers and have been seen as a promising analytical technology due to their simplicity.^118^

DNA-based electrochemical detection of HPV is the highly sensitive and specific method for the detection of HPV.^119^ In this case, the probe DNA is first immobilized on the electrode surface, then used to hybridise with the target complementary HPV DNA. The hybridization is detected by measuring the electrochemical signal response using any of the electrochemical techniques (such as SWV, DPV, EIS, *etc.*). The most important part of electrode fabrication is the ability to immobilize the probe DNA on the electrode surface.

### Immobilizing the HPV probe onto the electrode surface: some chemistry-inspired tricks

Immobilization of the HPV probe or the recognition element onto the electrode surface is the most critical step in the development of the immunosensor. If the probe (*i.e.*, antigen, antibody, DNA, or RNA) is properly oriented on the electrode surface, there is a high chance that the performance of the immune sensor would be improved. There are three chemistry-inspired tricks that one can adopt in immobilizing the probe onto the electrode surface, *viz*: (i) ionic bonding, (ii) covalent bonding (*i.e.*, amide bond creation), and (iii) self-assembly (or chemisorption) process (Table 2).^120^

**Table 2:** Different types of biosensors, their shortcomings and advantages^120^

Biosensor	Types	Advantages	Disadvantages	Solution
Electrochemical	Amperometric, potentiometric, conductimetric, impedimetric and voltametric	Excellent detection limits, faster detection, ease of fabrication, good resolution, linear output	Unstable current and voltage, limited/narrow range, short/limited shelf life, cross sensitivity with other gases	Design and employ electrocatalysts that are stable to minimize unstable currents and voltage
Optical	Surface plasmon resonance (SPR), fluorescence, bio/chemiluminescence, refractive index, Raman scattering, absorbance	High sensitivity, selectivity, cost-effectiveness, small size	Susceptible to physical change and interference from environmental effects	Use material that are stable in a wide range of temperatures
Thermal/calorimetric	Thermistors or thermopiles	Scalability, ease of use and ease of fabrication	Lack of specificity inn temperature measurements, long experimental procedures	Use high sensitivity thermocouple
Mass sensitive or gravimetric	Wave biosensor, surface acoustic, cantilever	Low cost and simplicity	Low sensitivity	Employ materials with high sensitivity to mass changes, such as piezoelectric materials or graphene
Piezoelectric	Surface acoustic devices, piezoelectric crystal	Fast detection, good frequency response, small size and high sensitivity	High temperature sensitivity, not suitable for static conditions, some crystal can dissolve in high humid environment and are water soluble	Maintain humid controlled environment and use water repellent coating

**Fig. 9:**
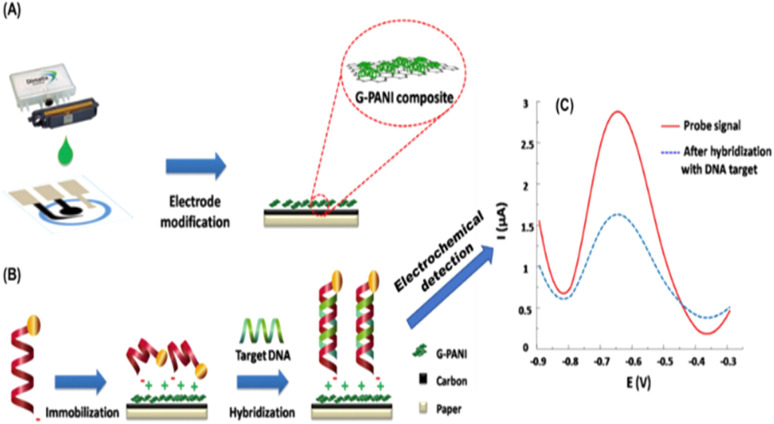
Schematic illustration of (A) electrode modification and (B) immobilization and hybridization steps of paper-based electrochemical DNA biosensor. (C) Square-wave voltammograms of immobilized AQ-PNA probe on G-PANI/SPCE before and after hybridization with an equimolar concentration of target DNA.^121^

The ionic bonding technique may be exemplified by the work of Teengam *et al.*^121^ who fabricated an HPV DNA sensor by first generating a positively charged amino group surface using polyaniline (PANI, emeraldine base) doped with camphor-10-sulfonic acid (CSA) that can coordinate with a negatively charged target DNA probe (Fig. 9). To obtain a negatively charged DNA probe, the authors modified the DNA probe with glutamic acid residues at the N-terminus to generate the negative charge, followed by end-capping with an acetyl group.

**Fig. 10:**
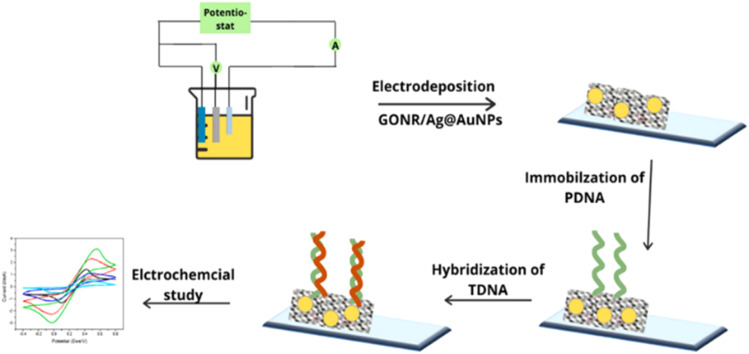
Stepwise representation of the encapsulation onto the ITO surface with GONR/Ag@AuNPs and probe DNA (PDNA) for electrochemical detection of the HPV target DNA (TDNA).^122^

Another way by which ionic bonding can be created is the use electrodeposition technique. Pareek *et al.*^122^ proposed an electrochemical biosensor comprising an indium tin oxide (ITO) coated glass modified with electrodeposited graphene oxide nanoribbons (GONR) and silver–coated gold nanoparticles (Ag@AuNPs) as the electrode for the immobilization of the probe DNA (PDNA) as a sensor for the target DNA (TDNA) of the HPV-16 (Fig. 10). The stability of the sensor was attributed to the electrostatic interaction between the negatively charged phosphate backbone of the PDNA and the positively charged nanomaterials (GONRs/Ag@AuNPs). The sensing activity was studied using CV and EIS. The proposed biosensor exhibited excellent sensitivity (0.54 mA aM^−1^) and low detection limit of 100 aM. The amide bond formation is the conventional technique for immobilizing antibody- or antigen-based probes. This technique is famous for immobilizing antigens and antibodies. However, since DNA contains amino terminal groups, it is also possible to adopt the amide-bonding technique. The process uses 1-ethyl-3-(3-dimethyl aminopropyl) carbodiimide hydrochloride (EDC) and *N*-hydroxy succinimide (NHS). The EDC/NHS activation approach possesses many merits which include high conversion efficiency, mild reaction conditions, excellent biocompatibility with little influence on the bioactivity of target molecules, and much cleaner products than other crosslinking reagents.^123–126^ The more water-soluble derivative of NHS-hydroxysulfosuccinimide (sulfo-NHS)/EDC has also been used as a molecular linker to immobilize the DNA probe on the graphite-based substrate.^110,127,128^ Alternatively, one can utilize 1-pyrenebutyric acid-*N*-hydroxysuccinimide ester (PBSE) as adopted by Asadi *et al.*^129^ for the detection of microRNAs (miRNA-21) which is a potential prostate cancer biomarker.

**Fig. 11:**
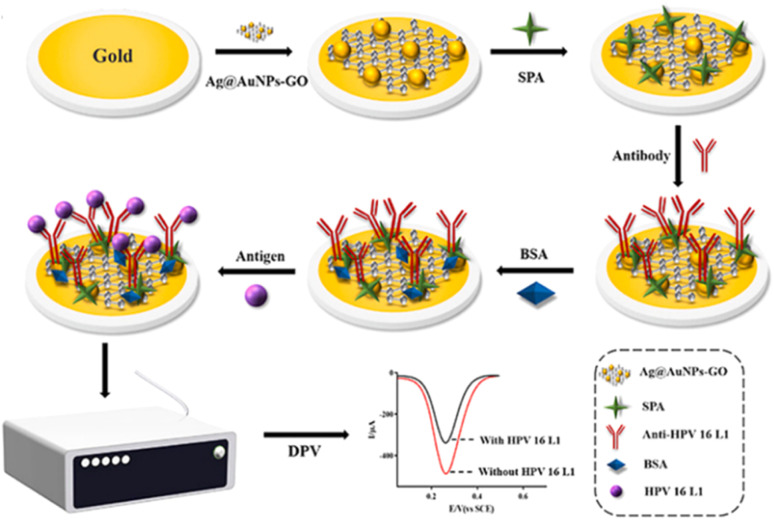
Stepwise encapsulation of Ag@AuNPs-GO, SPA and anti-HPV-16 L1 antibody onto the gold electrode surface for the electrochemical detection of the target antigenic HPV-16 L1 protein.^130^

The self-assembling process benefits from the strong affinity that exists between gold and sulphur-based molecules or thiolates that leads to the formation of a self-assembled monolayer (SAM). It is not surprising therefore that SAMs are formed on gold electrodes. Recently, Wang *et al.*^130^ encapsulated an anti-HPV-16 L1 monoclonal antibody on a gold electrode by using *Staphylococcal* protein A (SPA), instead of the conventional grafting technique that utilizes EDC/NHS, for the sensing of antigenic HPV-16 L1 (Fig. 11). SPA is found on the cell of the *Staphylococcus aureus* and is said to contain four active sites that can easily bind with the non-antigenic Fc receptor (*i.e.*, fragment crystallizable) portion of the immunoglobulin G (IgG), thus allowing the antigen-binding region (*i.e.*, fragment, antigen-binding) portion to be properly exposed to the surface for easy binding with the target antigens.

As shown in Fig. 6, several electrochemical techniques can be used for the detection of cancer biomarkers. The most important techniques are voltammetry (such as square wave voltammetry (SWV) and differential pulse voltammetry (DPV)) and Impedimetric technique. Impedimetric techniques have been proven to be a promising method for cancer biomarker detection due to their low excitation voltage, fast speed, and high sensitivity. They can be used for long-time, real-time, and on-site detection. Electrochemical impedance spectroscopy (EIS) is the most often used impedance method for electroanalytical detection^131–136^ is a high-sensitivity, low-cost, fast, label-free, and minimally invasive method for recording biological events. It uses an amplitude sinusoidal AC excitation signal, typically in the range of 2–10 mV, to determine the measurable resistance and capacitance characteristics of materials that adsorb the electrode surface. The low excitation voltage makes it become a safer detection technology for bioelectrochemical analysis systems that require long-term monitoring. As electrode heating is a problem that can cause changes in the biological microenvironment and damage to the electrodes, low excitation voltage does not generate a lot of heat, hence EIS is more suitable for long-term and real-time detection. Additionally, the EIS provides multiple parameters of the biosensing surface. Using a redox couple, typically a mixture of ferricyanide and ferrocyanide, the change in the charge transfer resistance (*R*_ct_) is obtained. Usually, the *R*_ct_ is inversely proportional to the rate of electron transfer. The double layer capacitance (CPE) and the *R*_ct_ describe the dielectric and isolation features of the electrode–electrolyte interface. The electrolyte resistance (*R*_s_) and the Warburg impedance (*Z*_w_) characterize the properties of an electrolytic solution and diffusion limitation for the redox probe to reach the electrode surface and do not affect electron transfer at the electrode surface. The detection in the broad frequency range (10^−4^–10^6^ Hz) makes the EIS strategy useful for diffusion analysis and for providing kinetics characteristics. Generally, at low frequencies (*f* < 1 mHz) the impedance is determined by the DC-conductivity of the electrolyte solution, and at higher frequencies (*f* > 100 kHz), the inductance of the electrochemical cell and connecting wires dominate the system.^137–139^

**Fig. 12:**
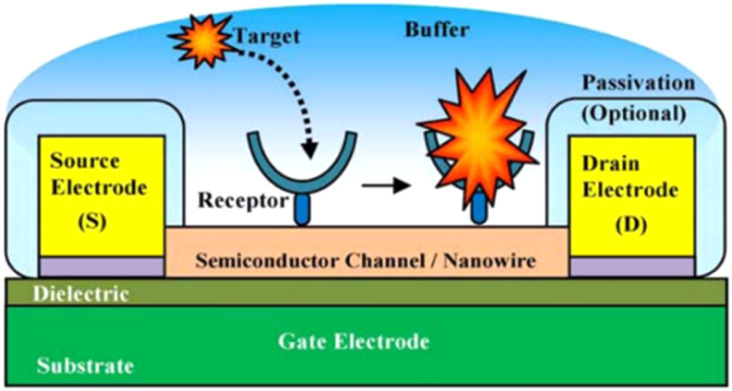
Experimental illustration of a nanoscale FET biosensor.^144^

Field-Effect Transistor (FET) based biosensors measure the conductivity of a channel (*i.e.*, region depleted of charge materials), between two electrodes (the source and drain) in FET (semiconductor) devices, see Fig. 12 for experimental illustration. Once the probe binds its analyte molecule, the electric field of its environment alters, thus producing a measurable change in such source-drain conductivity.

In recent years, several FET techniques have been reported for the sensitive and selective detection of HPV.^140–144^ For example, Aspermair *et al.*^140^ reported the use of rGO-FET for ultrasensitive and selective detection of HPV-16 E7 protein. The high performance of this sensor was attributed to attractive semiconducting characteristics of pyrene-modified rGO functionalized with RNA aptamer Sc5-c3. The aptamer-functionalized rGO-FET allows for monitoring the aptamer-HPV-16 E7 protein binding in real-time with a detection limit of about 100 pg mL^−1^ (1.75 nM). The authors successfully demonstrated the feasibility of this rGO-FET sensor for clinical application in point-of-care technology. HPV DNA and human telomerase reverse transcriptase (hTERT) mRNA are important biomarkers for cervical cancers because of their high levels of expression in cervical cancer cells, but little or no expression in normal cervical tissue. Some workers^141^ demonstrated the efficacy of a handheld Lab-on-Chip (LoC) device, based on an Ion-Sensitive Field-Effect Transistor (ISFET) sensor, in detecting cervical cancer from biopsy samples. The device was combined with loop-mediated isothermal amplification (LAMP) assays with the objective of amplifying the HPV DNA and hTERT mRNA.

Gao *et al.*^142^ fabricated a nanomaterial-based field transistor (FET) sensor made with the polyethylene glycol (PEG)-modified graphene device that exhibited real-time reversible detection of prostate-specific antigen (PSA) from 1 to 1000 nm in 100 mM phosphate buffer. Lu *et al.*^143^ also developed a complementary metal oxide semiconductor (CMOS)-compatible SiNW-FET biosensor fabricated by an anisotropic wet etching technology. They reported a rapid (<1 minute) detection of miR-21 and miR-205, with a low limit of detection (LOD) of 1 zeptomole (*ca.* 600 copies), as well as excellent discrimination for single-nucleotide mismatched sequences of tumor-associated miRNAs.

Compared with other traditional clinical diagnostic tools, the electrochemical techniques display sensitivity, simple operation, and rapid detection. The development of nanomaterials, antifouling coatings, and isothermal amplification technology has enabled a significant improvement in the performance of electrochemical biosensors, including sensitivity and specificity and sensor stabilities, *etc.* Nevertheless, to accomplish their application in clinical use, they require further improvement in several aspects, including sensor accuracy, miniaturization, and intelligence of instruments, and the assumption of long-term monitoring *in vivo*, which will provide a bright future for their diagnostic and prognostic clinical applications.

## Point-of-care (POC) approaches for HPV detection: current status

Screen-printed electrodes (SPE) are usually used for measurements carried out for research in areas such as medicine, pharmacy, food industry, agriculture, environment, or national security.^127,145^ Screen-printed electrodes can be fabricated, configured, and designed to be used in electrochemical applications for the detection and identification of drugs, pathogenic microorganisms, viruses, and protein biomarkers for diseases such as cancer, metabolic syndrome (MS) and for clinical analysis purposes in other to avoid human health problems. Moreover, they can be built as sensor arrays that allow the determination of multiple substances in parallel. Various studies that have been carried out using screen-printed electrodes and electrochemical analysis have shown that the material from which it is made – the working electrode (WE) exhibits a major role in the modulation of the electrochemical response. Hence the functionalization and modification of the working electrode by different bodies or elements to detect the target analyte is of utmost importance in its fabrication. Screen-plated electrodes are named after the element from which the working electrode is made, thus there are carbon, gold, platinum, palladium, or other metals. The screen-printed carbon seems to be the electrode of choice for researchers when it comes to developing fast and cost-effective methods to detect or quantify disease-inducing agents. A typical screen-plated electrode can be connected to an amplifier – potentiostat and subjected to various electrochemical measurements. It contains a working electrode (WE), an auxiliary or counter electrode (CE) and reference electrode (RE). There are some with a 4-electrode variation which comprises a working electrode, working sense, auxiliary/counter electrode, and reference electrode.

Lately, research for the development of biosensors has exploded, becoming a field of research for each type of biosensor, *i.e.*, DNA (deoxyribonucleic acid)-based sensors (genosensors), aptasensors, immunosensors, and enzymatic biosensors. In a recent review by Mincu *et al.*^146^ the authors reported that the aptamer-based sensors showed slightly better specificity and affinity for cancer-related biomarkers in comparison to antibodies-based sensors (immunosensors). In essence, screen-plated electrodes have come to the fore in the development of rapid *in vitro* diagnosis methods *via* their functionalization and immobilization with molecules like proteins, antibodies, antigens, enzymes, oligonucleotides, *etc.* in the development of these investigations.

**Fig. 13:**
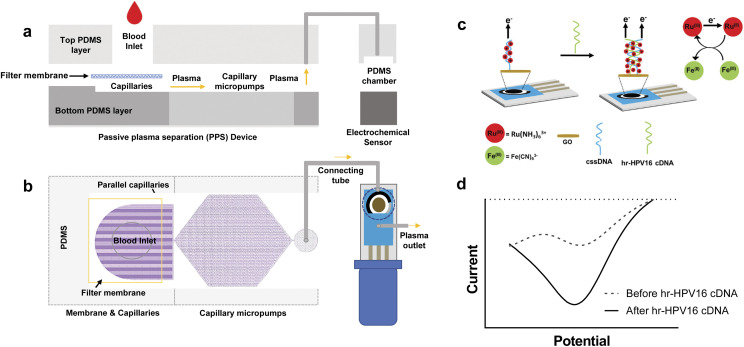
Overview and working mechanisms of IMEAC. Side view (a) and top view (b) of the IMEAC device that integrates two main modules: PPS for plasma isolation and an electrochemical biosensor. (c) Working principle of the electrochemical sensor for hr-HPV16 cDNA detection. (d) A sample graph extracted from IMEAC showing the presence of hr-HPV cDNA in the extracted plasma sample.^147^

The early diagnosis of cervical cancer as a vital factor for its successful treatment cannot be overemphasized. A current report by Keyvani *et al.*^147^ documented a novel integrated microfluidic electrochemical assay (IMEAC) that enables the detection of hr-HPV16-cDNA in an extracted plasma sample. The detection is achieved *via* graphene oxide (GO) modified screen-plated carbon electrode (SPCE) immobilized with cssDNA probe molecules that perceive the hr-HPV16 cDNA target (Fig. 13). The concentration range of hr-HPV cDNA in the plasma of cervical cancer is 1099 copies per ml while the authors reported a limit of detection (LOD) of 0.48 μM which is translated to ∼10^9^ copies per ml. And though the LOD obtained does not cover the clinical range, it is the first time work on the detection of hr-HPV DNA in plasma has been reported. They also believe that the IMEAC can be potentially used in identifying other biomarkers of cervical cancer like hr-HPV18 cDNA from plasma by using suitable probe molecules, thus enabling multiplexed measurement. It is envisaged that IMEAC (after some modifications to enhance its detection potencies and specificity) will be as a point-of-care diagnostic device for screening cervical cancer in remote areas.

Rawat *et al.*^124^ developed a flexible electrochemical DNA biosensor for the detection of HPV-16. The biosensor was fabricated using carbon coated SPEs which was initially coated with reduced graphene oxide (rGO) followed by the probe DNA (PDNA) immobilization. The novel CSPE/rGO/DNA bio-nanohybrids also possess a significant number of carboxyl groups for the efficient anchoring of HPV-16 PDNA. The sensor exhibited a LoD of ≈2 pM, it was found to be selective solely to HPV-16 target DNA with a shelf life and response time of 1 month and ≈15 s.

Bartosik *et al.*^148^ presented a SPE electrode that was assembled by the means of streptavidin-modified magnetic beads and a DNA capture probe to detect HPV16 DNA by using a digoxigenin label. The detection range of this biosensor was 1 pM to around 1 nM. Jampasa *et al.*^149^ used a screen-printed electrode (SPE) immobilized with an anthraquinone (AQ)-labeled pyrrolidinyl peptide nucleic acid (PNA) for identifying HPV L1 gene down to 4 nM. A screen-printed gold electrode was used as an electrochemical resistive DNA biosensor by Espinosa *et al.*^146,150,151^ to immobilize a DNA probe, complementary to human papillomavirus type 16 (HPV-16) sequence. The presence of a complementary sequence was detected by the change in resistance when the ssDNA is transformed in dsDNA due to the hybridization event. The detection limit of 2.39 nM was obtained. The authors also boast of a very short detection time out −750 μs, -in the resistive HPV-16/DNA/Au ensemble biosensor which makes it a new fast technique compared to the traditional EIS applied to DNA biosensors.

### Paper-based PoC platforms

The attractiveness of paper-based platforms in electrochemical sensing is because of their innate and interesting features which include their abundance, porosity, strong capillary action, disposability, lightness, flexibility, biocompatibility, eco-friendliness, and low cost.^152–154^ Paper-based electrochemical (bio)sensors have been employed to determine target analytes in highly variable matrices—soils, exhaust gases, waters, industrial sewage, cellular extracted DNA, blood, plasma, serum, urine, sweat, exhaled breath, and pharmaceutical capsules.^155–165^ Moreover, some of these (bio)devices made from paper-based substrates exhibit antifouling properties that are highly pursued to ensure the proper functioning of the devices in real biological matrices.

The introduction of electrochemical paper-based analytical devices (ePADs) has further intensified and opened a myriad of research in this field.^166^ It has launched a variety of fabrication procedures using different forms of paper materials like microfluidic PADs (μPAD).^167–169^ Yakoh *et al.*^170^ fabricated a 3D sequential fluid delivery platform on a microfluidic paper-based device (sePAD) which can store and transport reagents sequentially to the detection channel without the need for external power, thus eliminating the multiple-step reagent manipulation inherent to complex bioassays. The device comprises two components, which are an origami folding paper (oPAD) and a movable reagent-stored pad (rPAD) with two different configurations: the flow-through architecture, developed for continuous flow electrochemical measurements, such as chronoamperometry, and the stop-flow architecture, developed for non-convective electrochemical measurements, such as voltammetry. The 3D capillary-driven device served as an amperometric sensor for ascorbic acid determination; used for differential-pulse voltametric determination of serotonin and applied as an impedimetric immunosensor of α-fetoprotein detection.

**Fig. 14:**
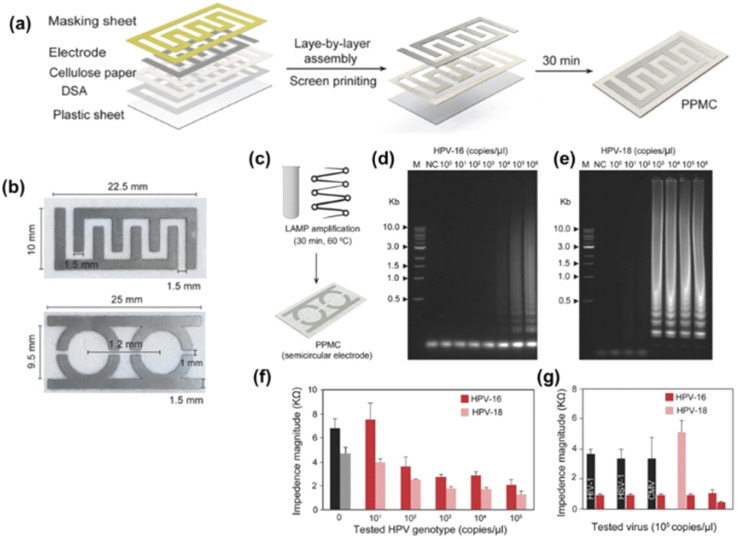
PPMC fabrication and characterization. (a) Fabrication of PPMC system using the screen-printing protocol coupled with layer-by-layer assembly. (b) Digital images of PPMC developed with interdigitated four-finger electrodes for single plex detection and two-semi-circular electrodes for multiplex detection. HPV nucleic acid detection and genotyping using PPMC with semi-circular electrodes (c) Schematic presentation of the developed PMMC-based nucleic acid assay for HPV DNA detection. Loop-mediated isothermal amplification (LAMP) technique was used to amplify the target HPV DNA using a set of four specific primers for each tested genotype by independent reactions and the formed amplicons were loaded on PPMC for impedance measurements. (d and e) Gel electrophoresis of LAMP amplification products generated from different concentrations of HPV DNA template. LAMP reaction was performed using tenfold serial dilutions of HPV DNA template (1 × 100 copies per microliter to 1 × 106 copies per microliter). M: 1-kb DNA ladder marker; NC: negative control (without target DNA template). (f) Impedance magnitude of LAMP amplicons prepared from different concentrations of target HPV templates at 8000 Hz and 1 V. For each concentration, the impedance magnitude was initially measured for LAMP amplicons of HPV-16 loaded on one of the testing zones and then for LAMP amplicons of HPV-18 loaded on the other testing zone. (g) Impedance magnitude of LAMP amplicons prepared from the target HPV-16 and nontarget viruses and genotype of human immunodeficiency virus-1 (HIV-1), herpes simplex virus-1 (HSV-1), and cytomegalovirus (CMV). Error bars represent the standard error of the mean calculated of at least three independent trials.^171^

Draz *et al.*^171^ developed paper–plastic microchip (PPMC) comprising three-layer hybrid substrates prepared of a cellulose paper substrate assembled with a transparent plastic sheet by double-sided adhesive (DSA), as seen in Fig. 14.

The fabrication process of PPMC is simple and leverages the advantages of the well-known layer-by-layer assembly and screen-printing protocols. The microchip with an upper surface made of cellulose paper and a lower back layer of plastic was adopted for electrical sensing of different targets, including liver and colon cancer biomarkers, and human papillomavirus (HPV). Coupled with loop-mediated isothermal amplification (LAMP), the developed PPMC electrodes were used for nucleic acid testing and genotyping of HPV. Two sets of primers, each comprising four specific primers, which are specific for two different genotypes of HPV-16 and HPV-18, were used for LAMP amplification. The formed LAMP amplicons in each reaction were simultaneously tested on PPMC designed with two detection zones (one was specified for HPV-16 and the other for HPV-18). The authors reported ladder-like amplicons, characteristic of LAMP was observed for both HPV-16 and HPV-18, confirming the specific amplification of the target HPV genotype. The loading of LAMP amplicons to the surface of PPMC resulted in a significant decrease in the impedance magnitudes measured at 8000 Hz and 1 V. The change in the impedance magnitude was inversely proportional to the tested concentration of the target HPV plasmid used in the samples of both HPV genotypes tested. Using this approach, the proposed PPMC can detect concentrations as low as 10^2^ copies per microliter and 10^3^ copies per microliter of HPV-18 and HPV-16, respectively.

Teengam, *et al.*,^121^ built an ePAD-based peptide nucleic acid biosensor for selectively detecting HPV-16. It was developed using an anthraquinone-labeled pyrrolidinyl peptide nucleic acid (acpcPNA) probe (AQ-PNA) and graphene-polyaniline (G-PANI). An inkjet printing technique was employed to prepare the paper-based G-PANI-modified working electrode. The paper-based electrochemical DNA biosensor was used to detect a synthetic 14-base oligonucleotide target with a sequence corresponding to human papillomavirus (HPV) type 16 DNA by measuring the electrochemical signal response of the AQ label using square-wave voltammetry before and after hybridization. It was determined that the current signal significantly decreased after the addition of target DNA. This phenomenon is explained by the rigidity of PNA-DNA duplexes, which obstructs the accessibility of electron transfer from the AQ label to the electrode surface. Under optimal conditions, the detection limit of HPV type 16 DNA was found to be 2.3 nM with a linear range of 10–200 nM. The performance of this biosensor on real DNA samples was tested with the detection of PCR-amplified DNA samples from the SiHa cell line.

A comprehensive review on colorimetric paper-based sensors was done by Carneiro *et al.*^172^ in which the authors set out to elucidate the application of paper as a substrate in sensor devices and the use of colorimetry for signal transduction and detection of cancer biomarkers. They surmised that improvements in the signal-amplification strategies have advanced and promoted the development of paper-based analytical devices (PAD) over the years; that the use of novel materials like nanoparticles as labels has increased sensitivity and provided clear signals based on color change. They, however, stated that reports on real-world applications of PADs for colorimetric detection of biomarkers, such as cancer, are still very limited. Thus, more research is needed to identify and address the challenges of this technique which include high limits of detection, insufficient specificity, poor stability, the need for multiplexing, and subjective interpretation of the results.

### Smartphone-based platforms

**Fig. 15:**
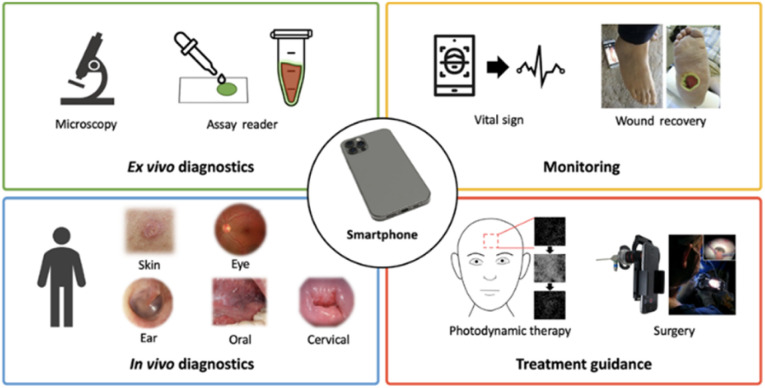
Smartphone-based imaging (SBI) SBI for various biomedical imaging applications grouped into four clinical workflows.^173^

The new technological era has ushered in a wave of having everything either in miniature or converted to portable accessories. An example is combining conventional biosensing technologies with handy, portable, and easy-to-carry mobiles. With the knowledge of the extreme societal penetration of smartphones and their common presence, mobile-sensing approaches offer significant advantages over traditional platforms. Synergistic use of sensing technologies with mobile technology enables the development of powerful portable platforms for various applications. Smartphone capabilities include cameras, touchscreens, networking, computation, 3D sensing, audio, and motion, in addition to commercial wearable peripheral devices that can be leveraged for biomedical imaging. And through the user-centred design of custom hardware and software interfaces, these capabilities have the potential to enable portable, easy-to-use, point-of-care biomedical imaging systems. Hunt *et al.*^175^ listed some smartphone-based imaging (SBI) systems which they categorized into four groups centred on their intended applications and clinical workflow: *ex vivo* diagnostic, *in vivo* diagnostic, monitoring, and treatment guidance (Fig. 15).

Naorungroj *et al.*^174^ developed a smartphone-assisted paper-based colorimetric assay using pyrrolidinyl peptide nucleic acid (acpcPNA) as a probe for the detection of HPV DNA. Dextrin-stabilized gold nanoparticles (d-AuNPs) were used as the colorimetric reagent. After the duplex formation between the acpcPNA probe and the HPV DNA target, the acpcPNA probe was depleted and the residual probe can cause different degrees of d-AuNPs aggregation, resulting in a detectable color change. The different color change before and after the introduction of the DNA target as a function of the DNA concentration was quantified by analysing the colour intensity using the smartphone. The authors reported that under optimal experimental conditions, the colorimetric DNA sensor displayed a linear range for the detection of HPV DNA in the range of 1–1000 nM with a detection limit of 1 nM. It showed high stability for up to 7 days when stored at 4 °C with the percentage decrease of the signal being less than 10%. For real samples application, the developed sensor was successfully applied to detect PCR-amplified HPV DNA from cell line samples. To enhance their capabilities, a high-resolution microscope can be attached to a camera-enabled mobile phone, which enabled both bright-field and fluorescence imaging.^175^ Prompt and reliable triaging of high-risk HPV cases could help offset severe pathology bottlenecks in resource-limited regions^176,177^ and circumvent geographical and/or socioeconomic barriers to effective cervical cancer screening. Visual inspection with acetic acid (VIA) is adopted as a rapid, inexpensive alternative to standard cytology (Pap smears); however, it suffers from high rates of false-negative and false-positive results. This has triggered the application of the smartphone as an assisted accessory to the normal use of naked-eye visual inspection. In a recent study carried out among HPV-positive women living with HIV (WLWH) in Western Kenya, Mungo *et al.*^178^ reported that digital images of the cervix were taken (WLWH of 25–49 years) using a smartphone by a nonphysician provider following visual inspection with acetic acid. These digital images were evaluated by three off-site colposcopists for quality and diagnostic utility and assigned a presumed diagnosis. Judging by the off-site expert colposcopists, the images were of good quality and had diagnostic utility. However, they observed low sensitivity for the diagnosis of CIN2+ from the cervical images compared with histopathology which if used for triage would result in substantial loss of opportunity for treatment of high-grade precancer in a high-risk population.

Champin *et al.*^179^ conducted a systematic review to identify studies on the usefulness of the smartphone in detecting uterine cervical lesions. They reported that several studies reveal that digital images taken with a smartphone after a visual inspection with acetic acid (VIA) or Lugol's iodine (VILI) may be useful for detecting CIN. The smartphone images clicked after a VIA were found to be more sensitive than those following the VILI method or the VIA/VILI combination and naked-eye techniques in detecting uterine cervical lesions. Therefore, the authors surmised that smartphones could be useful in the early detection of uterine cervical lesions and could be an alternative to colposcopy in countries with limited health resources. However, they noted, its sensitivity and specificity are still limited. Dufeil *et al.*^180^ set out to determine whether combined examination by the naked eye and digital VIA [D-VIA] and VILI [D-VILI] improves the detection of CIN2+ as compared to the conventional evaluation. The combination of both methods yielded a sensitivity of 92.3% and a specificity of 23.2%. Indeed, the combination of VIA/VILI and D-VIA/VILI seems to provide an increase in sensitivity, with an acceptable decrease in specificity. Although the authors advised a replication of the study with a larger sample size would be necessary to draw definitive conclusions. Nonetheless, they are convinced that the digital cervical image is useful for the diagnosis of CIN2+ lesions as their study represents the best available evidence to date that suggests that D-VIA/VILI may potentially improve cervical cancer screening.

Recently, some authors reported on studies aimed to provide an evaluation of available data for smartphone use in low-resource settings in the context of D-VIA-based cervical cancer screenings between the years 2015 and 2021.^181^ The available results to date show that the quality of D-VIA images is satisfactory and enables CIN1/CIN2+ diagnosis and that a smartphone is a promising tool for cervical cancer screening monitoring and for on- and off-site supervision, and training. The images obtained can be stored in a VIA image bank and used for training. Also, sharing real-time images with long-distance experts will improve the quality of work of healthcare providers. Although the evidence supports that D-VIA improves CIN2+ diagnostic performance, the use of smartphone applications is only considered a tool to minimize the subjectivity of the diagnosis. They further advised that a computer-assisted automated visual evaluation will be able to discriminate between normal and CIN and will likely significantly improve diagnostic accuracies, as well as allow see-and-treat approaches. Thus, low LIMC healthcare providers should focus on the implementation and development of smartphone-based screening programs using D-VIA, as it is proven to be acceptable and inexpensive, and it aligns with the WHO's effort toward the elimination of cervical cancer in the twenty-first century.

## Conclusions and future outlooks

In this review, the advantages, and shortcomings of the traditional and emergent tools for screening and testing of HPV infections and cancer have been highlighted. To achieve the WHO's ambitious 2030 goal (*i.e.*, every country ensuring that 70% of their women are screened using a high-performance test by the age of 35, and again by the age of 45) will require the adoption of two or more techniques based on nucleic acids and immunological techniques. Currently, the PCR-based methods dominate the clinical detection tools for HPV infection. However, some recent reports^182,183^ the authors indicated that the application of immunology-based methods are “under reconsideration” as the probable precise strategies for accurate and sensitive diagnosis of HPV infection and cervical cancer. The reason for this emerging development is that PCR-based methods “can only be used to detect HPV DNA and HPV types and cannot be used to accurately predict HPV-positive cancers”. Immunological methods based on antigens and antibodies are characterized by their high affinity, high specificity, and biocompatibility, thus important for the development of high-performance diagnostic tools for HPV infection and cervical cancer.

**Fig. 16:**
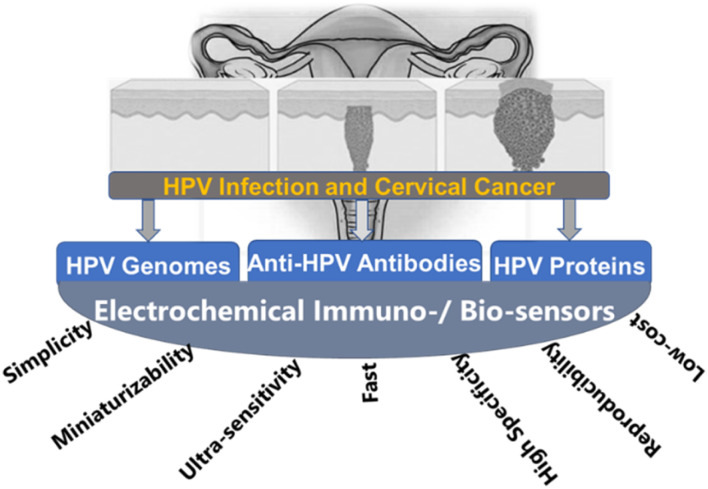
Schematic representation of the applicability of electrochemical methods for the detection of HPV infection and cancer through the detection of HPV genomes, anti-HPV antibodies, and HPV proteins.

Interestingly, the reconsideration of immunological methods for accurate HPV detection bodes well for fast realization of clinically relevant electrochemical immunosensing devices for HPV infection. It is interesting in the sense that electrochemical immuno-/bio-sensing techniques are characterised by simplicity (do not require highly skilled personnel), low-cost compared to other methods, highly sensitive, highly specific, easily to be miniaturize (portability), fast (quick analytical results) and highly reproducible. As shown in Fig. 16, three of the four traditional techniques for HPV detection (known for the low to moderate sensitivity and specificity) are already being reported in the literature but can be optimized to complement the other molecular biology-based methods.

**Fig. 17:**
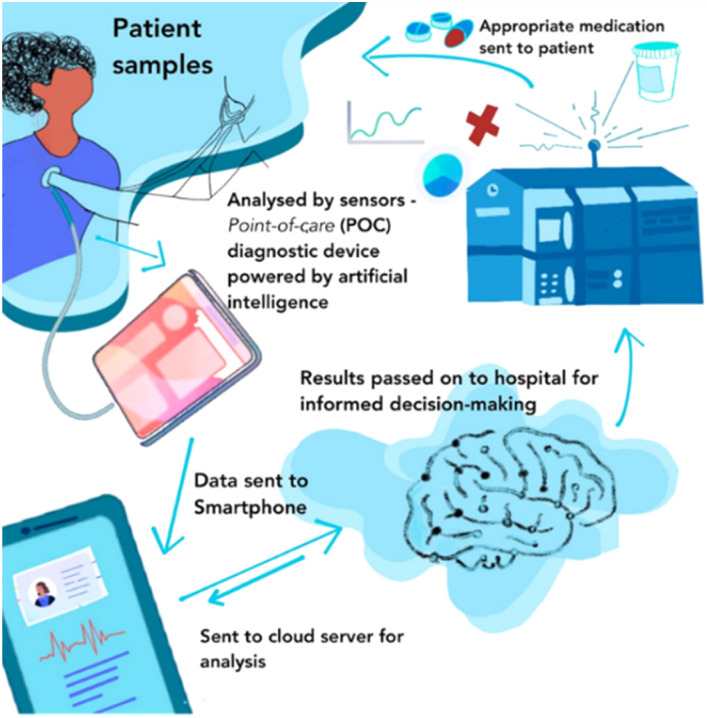
Schematic representation of the multi-disciplinary activities proposed for the prevention and control of HPV infection in the healthcare sector of resource-limited countries. The point-of-care diagnostic devices could be deployed for HPV infection and drug-monitoring, with dynamic spectrum broadband technology for an e-health that relies on secure data transmission from decentralized clinical benches (anywhere in the remote area) to the centralized laboratory, clinics, or hospitals.

In our view, the effective screening of HPV infection in resource-limited countries will require multi-disciplinary activities as depicted in Fig. 17. The access to digital camera is an important development in resource-limited societies that will positively impact on the effective screening of HPV infection. The patient's sample is analysed with the appropriate portable point-of-care diagnostic device (powered by artificial intelligence for increased sensitivity), the results (data) are sent to the cloud server *via* a smart phone, the results are passed on to the medical facility for informed decision-making that will allow for appropriate medication and/or further treatment.

Makower *et al.*^184^ in a report had stated that to improve the chances of having a technology that could transfer from an academic concept to a diagnostic system, scientists may have to consider a new diagnostic tool which overcome an “actual” problem/diagnostic request, and the current solution to it should be inadequate or expensive. The authors further surmised that such diagnostic tools if used for medical examination outside of medical labs (at home without skilled operators) must be precise, be user-friendly for untrained people, and have little risk of user confusion or harm if performed incorrectly. The diagnostic systems should be able to relay the test results automatically and safely to cloud-based systems, with appropriate maintenance of privacy. The prevalence of HPV infections remains high especially in developing countries despite tremendous efforts for HPV treatment and prevention. Currently, visual inspection with acetic acid (VIA) is adopted as a rapid, inexpensive alternative to standard cytology (Pap smear) is the main approach for the detection of early lesions of cervical cancer. The technique has however improved over the years, with the assistance of earlier cited technologies. In contrast, DNA testing for high-risk oncogenic HPV (subtypes 16 and 18) confers superior sensitivity (96–100%) and specificity (90–100%) along with greater clinical benefit when compared to cytology or VIA. This facilitates clinical counselling, follow-up examinations, thus improving treatment outcomes.

Electrochemical immuno-/biosensors offer an inexpensive, sensitive, simple, rapid, and portable alternative for viral detection that improves POC testing in ways that conventional methods have failed.^185^ These devices give access to early diagnosis of diseases in resource-limited settings, helping the world fight diseases efficiently. They can also be operated using different analytes. Antibody-based systems are a viable option for HPV point-of-care diagnostics, which is demonstrated in their longstanding popularity.^186^ Some antibody based diagnostic tools have made it from the laboratory to the consumer stage. An example is the OncoE6™ from Arbor Vita which is a lateral flow cervical cancer test device that detects the presence of E6 onco-proteins from high-risk types of human papilloma virus (HPV) types 16 and 18. However, the test takes 2.5 h, which is a significant length of time for a POC test.

The introduction of lectins and aptamers are contributing to POC testing due to their capacity for glycoprofiling. Jin *et al.*^187^ used an enzyme-linked lectin assay (ELLA) for detecting cervical intraepithelial neoplasia I (CIN I) and cervical cancer using serum immunoglobulins (Ig). The lectin-based assays such as ELLAs were found to have similar specificity and sensitivity to those seen in antibody-based ELISAs along with their ability to distinguish between glycosylation patterns. On the other hand, the authors reported that ELLAs were superior in discriminating CIN I and cervical cancerous cells from healthy cells while ELISAs were better able to differentiate between CIN I and cervical cancerous cells.

Non-invasive biomarkers have come up as could be “game-changers” in the early detection of cervical cancer. Some urinary proteins are being identified and used as biomarkers in early cervical cancer screening. This has also opened an avenue to self-sampling methods for early cervical cancer detection.^188–190^ Basak *et al.*^189^ identified a protein biomarker antigen (NCB-Ag) protein-phosphatase-1-gamma-2 (PP1γ2) specific to cervical cancer expressed in the urine sample. The antibody (Ab) specific to the NCB-Ag is attached to plasmonic Au NPs (≈5–20 nm) through a DTSP (3,3′-dithiodipropionic acid di(*n*-hydroxysuccinimide ester)) linker to form a composite Ab-DTSP-Au-NP. A localized surface plasmon resonance (LSPR)-based immunosensing method was used for the qualitative, reliable, and specific detection of the biomarker; thus, the Ab-DTSP-Au-NP composite undergoes a plasmonic shift after the interaction with NCB-Ag in urine samples.

In a very recent advancement, Chen *et al.*^190^ developed a novel photothermal triggered multi signal readout POC testing using a multifunctional vagina swab for HPV 17 E6 protein determination. The quantitative detection of target analyte was performed by using a portable fluorescence spectrometer and an inexpensive pressure meter in only 1 min with high sensitivity and accuracy. The composite probe SiC-CS@Ag (silicon decorated nanoparticles on chitosan-CS) triggered sensitive fluorescent quenching on a flexible fluorescence-temperature indicator (FLTI) and a remarkable pressure variation in pressure device under laser radiation. This bioassay realized sensitive target detection in the linear ranger from 10^−6^ ng mL^−1^ to 1 ng mL^−1^ with detection limits of 1.60 × 10^−6^ ng mL^−1^.

Machine learning and artificial intelligence, though still expensive cannot be left out in POC cervical cancer diagnostics. Pathania *et al.*^191^ developed an Artificial Intelligence Monitoring for HPV (AIM-HPV), that integrates low and high-tech solutions for DNA-based and POC cervical cancer screening. A disposable DNA extraction kit based on manual syringe operations was fabricated. This kit is a DNA-focused digital micro-holography platform monitoring for point-of-care HPV screening, with automated readouts driven by customized deep-learning algorithms. It incorporates microbeads designed to bind HPV 16 or 18 DNA targets and form microbead dimers. The authors reported the HPV DNA assay showed excellent sensitivity (down to a single cell) and specificity (100% concordance) in detecting HPV 16 and 18 DNA from cell lines. The deep learning approach was 120-fold faster than the traditional reconstruction method and completed the analysis in <2 min using a single central processing unit (CPU).

This review has attempted to capture the different point-of-care approaches for HPV detection, the challenges involved, and new advances and technologies to mitigate these challenges. As mentioned earlier some of these innovations can serve both diagnostic and prognostic purposes in HPV screening which can be integrated into primary health settings in LMICs as screen-and-treat models.

## Data availability

No primary research results, software or code have been included and no new data were generated or analysed as part of this review.

## Author contributions

Omobosede O. Fashedemi investigation, writing – original draft, figure drawing, Okoroike C. Ozoemena, investigation, visualization review, editing Siwaphiwe Peteni, investigation, visualization review, editing, Aderemi B. Haruna, reviewing, editing, investigation, Leshweni J. Shai, Aicheng Chen, Frankie Rawson, Maggie E. Cruickshank, David Grant, Oluwafunmilola Ola, reviewing, conceptualization, supervision, and Kenneth I. Ozoemena, conceptualization, reviewing, supervision.

## Conflicts of interest

There are no conflicts to declare.
